# Live Fluorescent Staining Platform for Drug-Screening and Mechanism-Analysis in Zebrafish for Bone Mineralization

**DOI:** 10.3390/molecules22122068

**Published:** 2017-11-27

**Authors:** Jung-Ren Chen, Yu-Heng Lai, Jhih-Jie Tsai, Chung-Der Hsiao

**Affiliations:** 1 Department of Biological Science & Technology College of Medicine, I-Shou University, Kaohsiung 82445, Taiwan; jrchen@isu.edu.tw; 2 Department of Chemistry, Chinese Culture University, Taipei 11114, Taiwan; lyh21@ulive.pccu.edu.tw; 3 Department of Bioscience Technology, Chung Yuan Christian University, Chung-Li 32023, Taiwan; cdhsiao@yahoo.com.tw; 4 Center for Biomedical Technology, Chung Yuan Christian University, Chung-Li 32023, Taiwan; 5 Center for Nanotechnology, Chung Yuan Christian University, Chung-Li 32023, Taiwan

**Keywords:** drug screening, bone mineralization, osteoclast, calcein, zebrafish

## Abstract

Currently, drug screening relies on cell-based experiments or on animal models to confirm biological effects. The mammalian system is considered too time-consuming, expensive and complex to perform high-throughput drug screening. There is a gap between in vitro cell-based models and the in vivo mammalian models. The zebrafish is an ideal model that could link preclinical toxicity screening with the drug development pipeline. Taking advantage of a highly conservative genomic, rapid development, large number of offspring, low cost and easy manipulation, zebrafish has been considered an excellent animal model for disease-based drug screening. In this study, zebrafish embryos were incubated with small molecular compounds that potentially affected bone mineralization in microplates. Two compounds of alendronate and dorsomorphin were used as positive and negative controls, respectively. The level of osteogenic mineralization was measured and quantified by using ImageJ software with fluorescent calcein-staining images. Among twenty-four tested compounds from the kinase inhibitor library, we identified two compounds, pentamidine and BML-267, which showed increased embryonic mineralization; while six compounds, RWJ-60475, levamisole HCL, tetramisole HCL, fenvalerate, NSC-663284, and BML-267ester, were inhibitory to bone mineralization. In addition, real time quantitative PCR (RT-qPCR) was performed to evaluate the biological pathways involved in bone metabolism at the molecular level. We confirmed that alendronate enhanced the level of bone mineralization by inhibiting osteoclast-related genes. In summary, our research established a simple method to screen potential bone metabolic drugs and to perform mechanism analysis for bone mineralization in vivo.

## 1. Introduction

Bone, a dynamic organ that serves mechanical and homeostatic functions, undergoes a continual self-regeneration called remodeling [1]. Bone remodeling is a process specified by a balance between bone formation by osteoblasts and bone resorption by osteoclasts [2]. An imbalance in bone remodeling contributes to several pathologic conditions, including osteosclerosis, osteopetrosis, and osteoporosis [3]. Osteosclerosis is a bone disorder characterized by abnormal thickening and progressive increase in bone mass because of increased number of osteoblasts. In contrast, osteopetrosis results from a primary decrease in osteoclastic function [4].

With the aging of the population, the cost of osteoporosis is an increasingly significant public health concern. In estimation, annual medical costs for osteoporosis in the U.S. range from $10 to $22 billion. There were also indirect costs including a reduction of quality of life and productivity [5,6,7,8,9]. Statistics showed that about 12 million people at the age of 50 years old will be afflicted with osteoporosis, while more than 40 million people will suffer from low bone mass. The incidence of osteoporosis is predicted to surge in the future. By 2020, 14 million cases of osteoporosis is estimated and more than 47 million cases of low bone mass. By 2025, the cost of osteoporosis may rise to nearly $25.3 billion annually in the U.S. [5]. In worldwide, osteoporosis has caused more than 8.9 million fractures annually and is estimated to affect 200 million people [6]. Osteoporosis is a huge personal and economic expense. In Europe, the incidence of osteoporosis was higher than all cancers [7]. It was greater than the occurrence of chronic non-communicable diseases, such as rheumatoid arthritis, asthma and high blood pressure-related heart disease [8].

Unlike other diseases, there are few agents that promote bone formation in patients with substantial bone loss [9]. Drugs that promote reabsorption were still limited in number [9]. Therefore, the development of a relatively simple, quick, effective animal platform, to screen for anabolic and catabolic therapeutic compounds and to evaluate the role of bone-related drug utility is urgent. Research on drugs that promote osteogenesis mainly uses cell culture or mouse model as drug screening platforms. For cell-based studies, for example, human mesenchymal stem cells (hMSCs) can be cultured in multi-well plates and adding a variety of drugs to see whether it can promote osteogenic differentiation by alkaline phosphatase staining or cell proliferative assay [10,11,12]. For animal-based studies, the thickness of the sections of the mice skull can be measured in order to compare the efficacy of the potential bone differentiation-promoting drugs [13].

The drug development process is considered costly and inefficient [14]. A crucial gap exists between in vitro and in vivo when validating novel drugs. The low throughput of mammalian models creates a major bottleneck to evaluate the numerous “hits” identified from cell-based screening. Zebrafish could enhance preclinical drug screening by its strategic placement between cell-based and mammalian models along the drug development pipeline. In addition, the availability of a large number of zebrafish embryos allows drug screening to be performed in microplates, which makes automation in a high-throughput manner possible. With lower maintenance cost and less space required for a zebrafish facility compared to a mammalian facility, it is more cost-effective to employ zebrafish for early preclinical drug screening. 

The many advantages of zebrafish include rapid embryonic development, large number of offspring, low cost for maintenance and similar bone histological structure make zebrafish as an ideal model for bone-related studies [15]. The rapid embryonic development makes the observation efficient and accurate. Compared to mammalian models, zebrafish is easily manipulated for generating transgenics by cytoplasmic rather than nuclear injection [16]. They also greatly speed up disease-based research in clinical studies by robotic microinjection of transgenic materials into zebrafish embryos to accelerate the process of genetic manipulation and reduce the need for manpower [17]. In this study, we have developed a rapid screening of anti-osteoporosis drugs in zebrafish. Furthermore, calcein was adapted as a vital dye for bone mineralization evaluation in vivo. The stained area on the vertebrate column was quantitated to evaluate the impact of the drugs on bone mineralization. When the candidate compounds were evaluated, molecular analysis continued to demonstrate the effects of drugs on osteoblasts, osteoclasts and calcium ions in the regulation of mineralization pathway, which made the validation complete.

## 2. Results

### 2.1. Optimization of Small Molecular Screening Platform in Zebrafish with Positive and Negative Control Compounds

**Figure 1:**
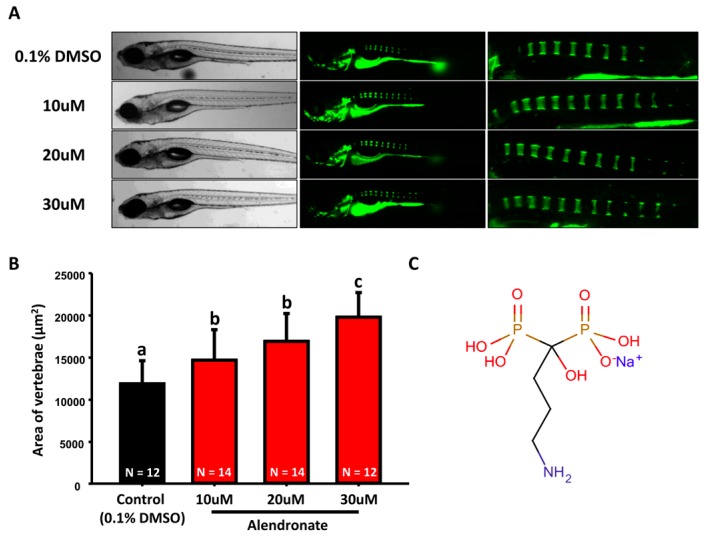
**Evaluation of mineralization in alendronate-treated zebrafish.** (**A**) The gross morphology of zebrafish aged at 7 dpf which have been treated with different concentrations of alendronate (10, 20, and 30 µM, left bright-filed panel) from 3 dpf onwards. Calcein staining on control and alendronate-treated embryos at 7 dpf (right green fluorescent panel); (**B**) Quantification of mineralization degree by detecting the fluorescence intensity at the area of centrum form ring in the notochord. (values are mean ± SD; tested by one-way ANOVA pairwise comparison; N = fish number; Different letters indicate significant differences) (**C**) Chemical structure of alendronate.

Previous studies have shown that alendronate promotes bone formation with a less anisotropic microstructure in mouse and rat models [15,18]. It also served as a potential treatment for patients who suffered from bone loss [19,20]. To investigate the effect of alendronate on the embryonic skeletal development, 3 dpf (day-post-fertilization) embryos were treated with alendronate at different concentrations (10, 20, and 30 µM) and compared to 0.1% DMSO control group by 7 dpf as an endpoint. We first evaluated the vertebrate column mineralization level of 7 dpf embryos with calcein staining. We observed that the vertebrate column mineralization area of alendronate-treated larvae was significantly increased compared to control group (Figure 1A,B). The structure of alendronate is shown in Figure 1C.

**Figure 2:**
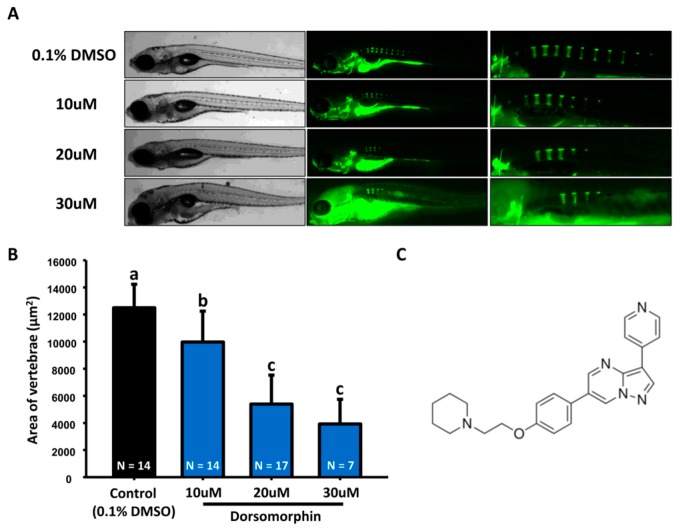
**Evaluation of mineralization of the vertebrate column in dorsomorphin-treated zebrafish.** (**A**) The gross morphology of zebrafish aged at 7 dpf which have been treated with different concentrations of dorsomorphin (10, 20, and 30 µM, left bright-filed panel) from 3 dpf onwards. Calcein staining on control and dorsomorphin-treated embryos aged at 7 dpf (right green fluorescent panel; (**B**) Quantification of mineralization degree detecting the fluorescence intensity at the area of centrum form ring in the notochord. (values are mean ± SD; tested by one-way ANOVA pairwise comparison; N = fish number; Different letters indicate significant differences) (**C**) Chemical structure of dorsomorphin.

Dorsomorphin, whose structure is shown in Figure 2C, inhibits Smad-dependent BMP (Bone morphogenetic protein) signaling, which has been shown to be a key promoter during osteogenesis [11,21]. We hypothesized that dorsomorphin may serve as a negative regulator for bone mineralization. As the data showed, when we treated zebrafish embryos with dorsomorphin at various concentrations (10, 20, and 30 µM) from 3 dpf onwards, we found that dorsomorphin caused decreased mineralization of vertebrate column in zebrafish larvae in a dose-dependent manner (Figure 2A,B). Combined data collected from alendronate and dorsomorphin, we concluded the biological effect of both compounds on zebrafish bone mineralization is well consistent with the phenotype observed in their mammalian counterparts.

### 2.2. Rapid Screening on Small Molecular Library

**Figure 3:**
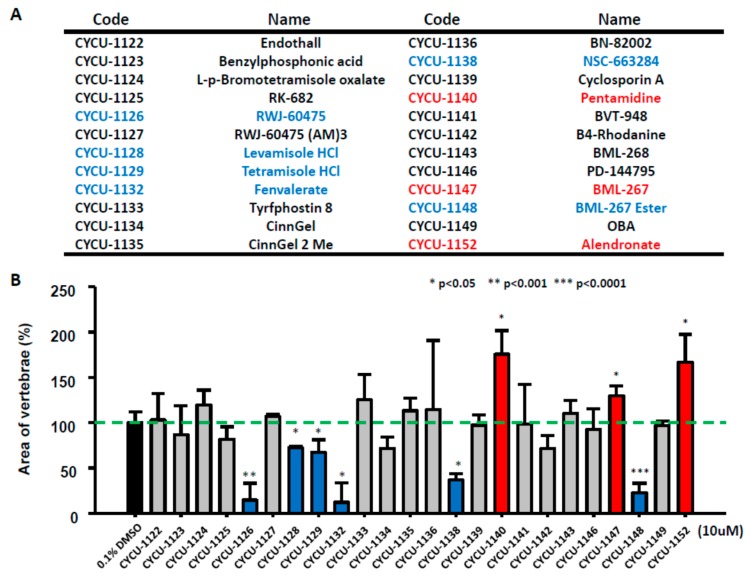
**Small molecular screening on bone development.** (**A**) List of small molecules in the library. (**B**) Relative mineralization level of zebrafish embryos after treating with different compounds at 10 µM. Zebrafish embryos were treated with 10 µM of each compound from 3 dpf onwards. The quantification was determined with fluorescence microscopy within vertebrate column area after calcein staining. The blue column showed decreased mineralization after treating the specific compound; whereas red showed increased. (values are mean ± SD; * *p* < 0.05; ** *p* < 0.001; *** *p* < 0.001; tested by student’s T test with 0.1% DMSO control group).

**Figure 4:**
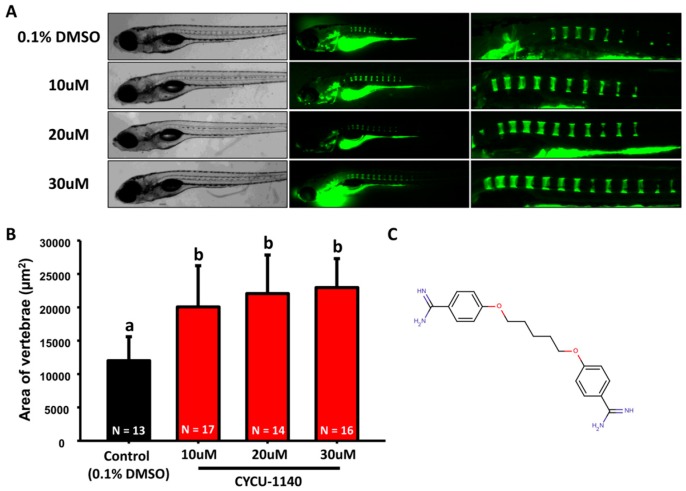
**Increase of mineralization in pentamidine-treated zebrafish.** (**A**) The gross morphology of zebrafish aged at 7 dpf which have been treated with different concentrations of pentamidine (10, 20, and 30 µM, left bright-filed panel) from 3 dpf onwards. Calcein staining on control and pentamidine-treated embryos at 7 dpf (right green fluorescent panel); (**B**) Quantification of mineralization degree detecting the fluorescence intensity at the area of centrum form ring in the notochord. (values are mean ± SD; tested by one-way ANOVA pairwise comparison; N = fish number; Different letters indicate significant differences) (**C**) Chemical structure of pentamidine.

**Figure 5:**
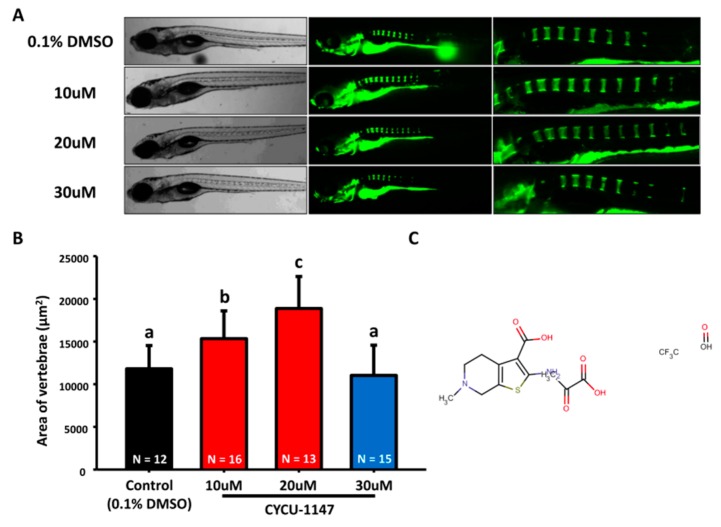
**Increase of mineralization in BML-267-treated zebrafish.** (**A**) The gross morphology of zebrafish aged at 7 dpf which have been treated with different concentrations of BML-267 (10, 20, and 30 µM, left bright-filed panel) from 3 dpf onwards. Calcein staining on control and BML-267-treated embryos at 7 dpf (right green fluorescent panel); (**B**) Quantification of mineralization degree detecting the fluorescence intensity at the area of centrum form ring in the notochord. (values are mean ± SD; tested by one-way ANOVA pairwise comparison; N = fish number; Different letters indicate significant differences) (**C**) Chemical structure of BML-267.

Inspired from the data of alendronate and dorsomorphin, we aimed to evaluate more compounds that might affect bone mineralization in zebrafish. We selected alkaline phosphatase inhibitor-like compounds in our chemical library. Zebrafish embryos aged at 3 dpf were treated with 10 µM of each compound, later the calcein staining was performed by 7 dpf. Compared to control group (in 0.1% DMSO), 12 out of 24 compounds showed embryonic toxicity. In addition, two compounds increased embryonic mineralization, while six compounds were inhibitory to bone mineralization (Figure 3A,B). Two compounds that caused increased vertebral area are pentamidine and BML-267. As a positive control, alendronate was also included for comparison. For pentamidine, zebrafish embryos aged at 3 dpf were treated with different concentrations and the mineralized areas were evaluated with calcein staining by 7 pdf (Figure 4C). The result showed that significant increase of bone mineralization was observed within zebrafish vertebral region (Figure 4A,B). Moreover, zebrafish treated with 10 µM pentamidine showed more pronounced mineralization than 30 µM treatment of alendronate. Therefore, we hypothesized that pentamidine is more potent to promote bone mineralization than alendronate. Next, we examined the mineralization effect on BML-267 (Figure 5C). Consistent with the increasing vertebral area observed at 10 µM of BML-267, the mineralization level of embryonic zebrafish was induced at 10 and 20 µM of BML-267 (Figure 5A,B). Interestingly, we have noticed an inhibitory effect of BML-267 at higher dose of 30 µM. The inhibitory effect of bone mineralization is not due to growth retardation in high BML-267 concentration, since the embryonic development seems normal from calcien staining. Further studies are required to clarify the inhibition phenomenon.

**Figure 6:**
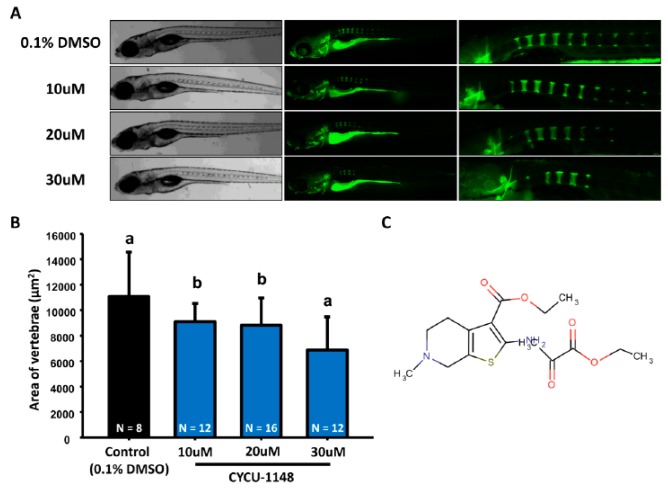
**Decrease of mineralization in BML-267ester-treated zebrafish.** (**A**) The gross morphology of zebrafish aged at 7 dpf which have been treated with different concentrations of BML-267 ester (10, 20, and 30 µM, left bright-filed panel) from 3 dpf onwards. Calcein staining on control and BML-267 ester -treated embryos at 7 dpf (right green fluorescent panel); (**B**) Quantification of mineralization degree detecting the fluorescence intensity at the area of centrum form ring in the notochord. (values are mean ± SD; tested by one-way ANOVA pairwise comparison; N = fish number; Different letters indicate significant differences) (**C**) Chemical structure of BML-267 ester.

Among the six compounds tested, RWJ-60475, levamisole HCL, tetramisole HCL, fenvalerate, NSC-663284, and BML-267ester showed the most inhibitory effect on mineralization (Figure 3B). A gradient of BML-267ester (Figure 6C) was chosen for validation of mineralization after treatment. As we expected, BML-267ester showed a dose-dependent decrease at mineralization level in embryonic zebrafish (Figure 6A,B).

### 2.3. Zebrafish Embryos are A Potential Drug-Screening and Mechanism-Analysis Platform for Bone Mineralization

**Figure 7:**
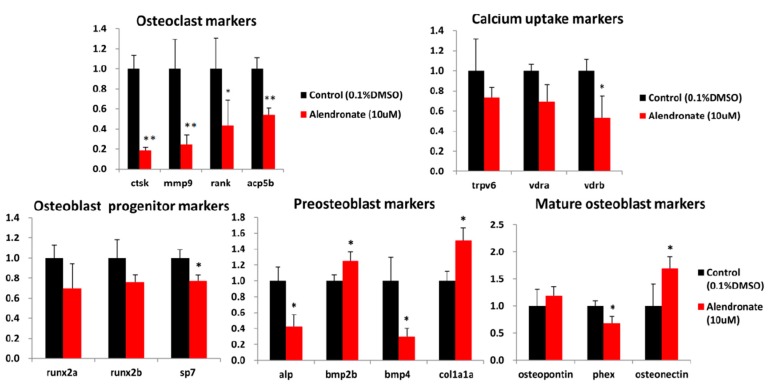
**Relative expression level of osteogenesis-related genes in alendronate-treated zebrafish.** Total RNA was extracted from alendronate-treated embryos aged at 7 dpf and the relative expression levels of marker gene were measured by RT-qPCR. (N = 10; values are mean ± SD; * *p* < 0.05; ** *p* < 0.001; tested by student’s *t* test with 0.1% DMSO control group).

Two processes are involved in bone formation: osteoblastic differentiation and osteoclastic inhibition. During osteoblastogenesis, mesenchymal stem cells (MSCs) are induced into osteoblasts, which proliferate and maturate into mineralized bone cells [22]. We next selected several genes that associated with bone development, and validated marker gene expression by RT-qPCR to confirm the affecting osteogenesis processes. Marker genes tested in this study are osteoclast-associated markers (*ctsk*, *mmp9*, *rank*, and *acp5b*), calcium absorption-related markers (*trpv6*, *vdra*, and *vdrb*), osteoblast progenitor markers (*runx2a*, *runx2b*, and *sp7*), preosteoblast markers (*alp*, *bmp2b*, *bmp4*, and *colla1a*) and mature osteoblast markers (*osteopontin*, *phex*, and *osteonectin*). Compared with the control group, *ctsk*, *mmp9*, *rank*, and *acp5b* were significantly downregulated in alendronate treated embryos (Figure 7). Our results suggested that osteoclast-related pathway was generally inhibited when zebrafish embryos treated with alendronate. On the other hand, *bmp2b* and *col1a1a* were upregulated, which were consistent with increased bone formation seen in the clinical settings [23]. In addition, *osteonectin* (also known as *sparc*, is a glycoprotein that secreted by osteoblasts that binds calcium to initiate mineralization and promoting mineral crystal formation) was significantly upregulated, which has been reported to link to bone mineralization [24]. The major calcium uptake channel *trpv6* [25,26,27] show no significant change in alendronate treated embryos, suggesting the increased level of mineralization is not contributing by the elevation of external calcium uptake. *Phex* is a transmembrane endopeptidase, thought to be involved in bone and dentin mineralization and renal phosphate reabsorption, showing un-expected downregulated in alendronate treated zebrafish embryos. In addition, the downregulated of *alp* and *bmp4* in alendronate treated zebrafish embryos (showing elevation of mineralization level) is also unexpected. Currently, we do not know why *phex*, *alp* and *bmp4* gene expression downregulated in alendronate treated zebrafish embryos. More studies should be addressed in the future to clarify this observation. Taken together, our data suggested alendronate triggered bone formation in zebrafish embryos through inhibition of osteoclast-associated signaling, and activated bone mineralization through bmp2b-osteonectin pathway.

## 3. Discussion

The bone formation process in zebrafish is like their mammalian counterparts, which consists of processes of intramembranous ossification and endochondral ossification [28]. The process of intramembranous ossification begins with the formation of interstitial stem cells in the cartilage, then the bone forms in the cartilage, and finally the cartilage is replaced by the bone. The flat bones that compose most of the skull are generated by intramembranous ossification. The intramembranous ossification process does not undergo the formation of cartilage. Rather, interstitial stem cells directly differentiate into osteoblasts, secrete extracellular matrices for mineralization, and finally form bones [29]. Bone formation processes are conserved in mammalian and fish systems, during both intramembranous and endochondral ossification within the craniofacial skeleton [30,31]. The molecular mechanisms underlying the bone physiology are similar between two species [32]. However, there are several differences in which zebrafish bone developments vary from those of rodents. The initial ossification events in the axial skeleton appeared at late larval stages, from 7 to 9 dpf, which were initially through an acellular rather than an endochondral mechanism [30]. Spinal cord development in zebrafish is also different from mammals, in that the spinal cord of a mammalian embryo was formed by intramembranous ossification during early development, while in zebrafish it was formed through endochondral ossification, without the formation of cartilage skeleton. As early as seven day-post-fertilization, part of the notochord began to mineralize, and formed the vertebrae directly. In addition, regional differences in the response of the zebrafish skeleton to BMP have been documented [33,34]. The ossification processes within craniofacial region are similar to those in mammals. Osteoblasts and osteocytes both existed in larval zebrafish [30]. Therefore, larval zebrafish bones contained the sufficient and necessary cells for both bone formation and resorption activity. Compared to their high vertebrate counterparts, the translucent zebrafish embryos allow for direct observation of the mineralization process in vivo.

Generally, during preclinical drug mining, candidate validation and toxicity analysis is performed with in vitro cell-based approaches as first step. Later, by using the knowledge from in vitro assays, in vivo pharmacology and toxicology will be conducted and tested in a mammalian model [28,29]. In vivo screening has been established for bone anabolic compounds in zebrafish by using non-vital dye of alizarin red [35]. The application of zebrafish on bone-related studies has been applied to some clinically well-known drugs, such as vitamin D3 (cholecalciferol), calcitriol, parathyroid hormone Teripalatide (Teriparatide), etc., which promote bone mineralization [35]. Previous study has proposed a rapid screening protocol by using calcein for the antagonist in Bone Morphogenetic Protein (BMP) signaling and found dorsomorphrin play an inhibitory role on bone mineralization in zebrafish larvae [11]. In this study, we examined more compounds and improved the methodology for calcein staining and performed an image-based rapid screening of zebrafish embryos for bone mineralization. Compared with alizarin red staining used by Fleming et al., 2005 [33], the live fluorescent calcein-staining labels calcified skeletal structures, and it is a more sensitive and inclusive method for visualizing skeletal structures in developing zebrafish embryos. Recent study pointed out zebrafish operculum is a good target to assess osteogenic bioactivities [36]. In the operculum-based drug screen system, embryos were treated with chemicals from 3 dpf onwards and detect the mineralization level of operculum by alizarin red at 6 dpf. Although the timing and protocol between vertebrate column-based (this study) and operculum-based methods [36] are similar, however, the calcein dye used in our study is a vital dye and no need to sacrifice fish at the end of experiment. This vital dye method makes it is possible to detect biological events of zebrafish at phenotypic or molecular levels after the experimental endpoint.

As a pilot screening, we identified two compounds that can increase embryonic mineralization, while six compounds can inhibit bone mineralization (Figure 3). Although we processed the screening manually, our quantification screening with calcein staining indeed facilitated the process and diminished labor of handling. The contextual platform we provided may have the potential to scale up automation in the future. Furthermore, by using alendronate as a validation compound in our system, we demonstrated that phenotypic endpoints could corroborate with molecular tools such as RT-qPCR to characterize the mechanism of the candidates in a conceptual framework of cause-and-effect, with verifications from known molecular interactions and phenotypic anchoring. We examined several marker genes that are associated with bone development for the activities of these genes: osteoclast-associated markers, calcium absorption-related markers, osteoblast progenitor markers, preosteoblast markers, and mature osteoblast markers, to confirm the affected osteogenesis processes in alendronate treated embryos. The results suggested to us that the osteoclast-related pathway was generally inhibited. On the other hand, *bmp2b* and *col1a1a* were upregulated, which is consistent with increased bone formation seen in the clinical settings [23]. In this study, we have provided in vivo evidence to show alendronate triggers bone formation through inhibiting on osteoclast-associated signaling, and activates bone mineralization through a bmp2b-osteonectin pathway. By using a similar approach, we believe that the biological mechanism for other bone mineralization-promoting or -inhibiting drugs can be elucidated in future study. Taken together, our results demonstrate that zebrafish is a potential drug-screening and mechanism-analysis platform for bone mineralization in vivo. It has the advantage of in vitro cell-based assays allowing for quick screening and affordability and reliability for identifying drug candidates. In addition, the advantage of whole animal experiment is reliability for predicting drug candidate performance and potential toxicity in vivo.

## 4. Materials and Methods

### 4.1. Zebrafish Maintenance

Fish were maintained according to the standard method described in the zebrafish book [37]. AB strain fish was feed with dry food and *Artemia salina* twice a day. The night before breeding, males and females were separated into different tanks at a ratio of 1 male to 2 females. At the beginning of next light cycle, the males and females were allowed to mate in the same tank. Embryos were collected and cultured in petri dishes containing E3 water (5.0 mM NaCl, 0.17 mM KCl, 0.33 mM CaCl_2_ and 0.33 mM MgSO_4_) at 28 °C under a14 h on/10 h off light cycle. All animal experiments in this study were performed in accordance with the guidelines issued by the animal ethics committee of Chung Yuan Christian University (Number: CYCU10014).

### 4.2. Small Molecular Library

The CYCU-1120~1152 chemical library (Chung Yuan Christian University, Department of Bioscience Technology) was purchased from Enzo Life Sciences, Inc. (SCREEN-WELL^®^ Kinase Inhibitor library, BML-2832, Farmingdale, NY, USA) as the source of small molecules for the experiment. The active compounds in the library, alendronate (Enzo, PR-123, Farmingdale, NY, USA) and dorsomorphin (Sigma-Aldrich, P5499, Missouri, MO, USA) were dissolved in 100% dimethyl sulfoxide (DMSO) to 10 mM stock and finally were diluted with E3 water to working solution (at a range from 10 to 30 µM) for later experiment.

### 4.3. Calcein Labeling 

Calcein staining is a fluorescent dye used for detecting calcium as an index of mineralization [38]. Due to considerations on the bone developmental progress, early observation with calcein staining would be insufficiently clear to show the mineralization. Zebrafish embryos aged at 7 dpf were immersed in 1% calcein working solution (Sigma-Aldrich, C0875, Missouri, MO, USA) for 10 mins and later washed with E3 water three times (each time 3 mins) to remove unbound dye. The fluorescent signals of the calcein-stained vertebrate column in the spinal region of zebrafish was observed at magnification of 10X under fluorescence microscopy (SMZ1500, Nikon, Tokyo, Japan) equipped with long pass green filter (excitation 480 ± 40 nm; emission 510 nm).

### 4.4. Small Molecular Screening

Synchronized embryos aged at 3 dpf were collected and arrayed by pipette. Twenty embryos per well were relocated into a 12-well plate for individual compound test. Small molecular compound stocks at 10 mM were diluted with E3 water to working concentrations ranged from 10 to 30 µM, and then added to embryos from 3 dpf onwards. According to our optimization on small molecular treatment for bone mineralization, fish could be treated as early as from 3 dpf onwards. The later the treatment was given, the less effect could be observed; however, early treatment might cause lethality. The toxicological effects of zebrafish were observed under the microscope from 3 dpf onwards until 7 dpf. During the screening period, dead fish were removed frequently and replaced the diluted compound to keep a more stable concentration of compound tested. 

### 4.5. Real Time Quantitative PCR (RT-qPCR) 

Total messenger RNA from zebrafish embryos aged at 3 dpf were extracted by the RNAZol^®^RT (Invitrogen), and quantified with NanoDrop (Thermo Scientific, Waltham, MA, USA). RevertAid first cDNA synthesis kit (K1622, Fermentas, Waltham, MA, USA) was used to synthesize first-strand cDNA from total zebrafish RNA according to the manufacturer’s instructions. RT-qPCR was performed using iQ SYBR Green Supermix (Bio-Rad Laboratories, Hercules, CA, USA) on a Bio-Rad iCycler using *β-actin* as control, and data were analyzed using the ∆∆Ct method [39]. The primer sequences used to perform quantitative real-time PCR are listed in Table 1.

### 4.6. Image Processing

Embryos and larvae were anesthetized with 0.16% tricaine methane sulfonate (MS222) in E3 water. Water was dispensed and the paralyzed embryos and larvae were moved into a plate containing 4% methylcellulose. Fish were orientated to an anterior-to-left and dorsal up position to facilitate image capture and comparison. Images were captured under florescence microscopy (SMZ1500, Nikon, Tokyo, Japan) mounted with a cool CCD (Evolution VF, Media Cybernectics, Rockville, MD, USA). The calcein stain-positive area of the vertebrate column was selected as region of interesting (ROI). The captured images were converted into binary black and white images by ImageJ software (version 1.44, https://imagej.nih.gov/ij/). Since the signal-to-noise ratio is high for calcein staining, we routinely calculate the calcein stain-positive area by using auto-threshold settings in ImageJ.

### 4.7. Statistical Analysis

The data was expressed as mean ± SD and the potential difference was tested by Student’s-*t* test or one-way ANOVA pairwise comparison. The significance level was set at 5% (*p* < 0.05). The letter indicated different significance in figures; a, *p* < 0.5; *b* < 0.01; *c* < 0.005.

## Figures and Tables

**Table 1:** Primers used for RT-qPCR.

Gene Name	Symbol	Forward Primer	Reverse Primer
Osteoclast markers			
cathepsin K	*ctsk*	GGACTCAATCACTATCACT	AGAACAAGACATCTAAGACA
matrix metallopeptidase 9	*mmp9*	TCGGCCTACCAAGCGACTT	TCATGTGAATCAATGGGCACTC
receptor activator of nuclear factor kappa-B ligand	*rank*	GCACGGTTATTGTTGTTA	TATTCAGAGGTGGTGTTAT
acid phosphatase 5b	*acp5b*	GCTGCTGCTAACAAACAAT	GACCAACCACGATGACAA
Calcium uptake markers			
transient receptor potential cation channel subfamily V member 6	*trpv6*	GATCGCAATGACATAATG	CTCCATCACTCTTAGAAG
vitamin D receptor a	*vdra*	CTTCAGACTCATTCAACCAT	GATACATCATCAGCAGATTACT
vitamin D receptor b	*vdrb*	CTCATCAGACTCCTTCAG	TACATCATCAGCAGGTTAC
Osteoblast progenitor markers			
runt-related transcription factor 2a	*runx2a*	GACGGTGGTGACGGTAATGG	TGCGGTGGGTTCGTGAATA
runt-related transcription factor 2b	*runx2b*	CGGCTCCTACCAGTTCTCCA	CCATCTCCCTCCACTCCTCC
sp7 transcription factor 7	*sp7*	GGCTATGCTAACTGCGACCTG	GCTTTCATTGCGTCCGTTTT
Preosteoblast markers			
alkaline phosphatase	*alp*	CAAGAACTCAACAAGAAC	TGAGCATTGGTGTTATAC
bone morphogenetic protein 2b	*bmp2b*	CGGCTCCTACCAGTTCTCCA	CCATCTCCCTCCACTCCTCC
bone morphogenetic protein 4	*bmp4*	TTGTGCTGTGCATGTTTGAA	GGTCGCTTGGCTATGTGTTT
collagen type II, alpha 1 (cartilage collagen)	*cola1a*	CTGTGCCAATCCCATCATTTC	ATATCGCCTGGTTCTCCTTTC
Mature osteoblast markers			
osteopontin	*opn*	GCCTCCATCATCATCGTA	AATCACCAAGCACCAGTA
phosphate regulating endopeptidase homolog, X-linked	*phex*	GAGAATGAATGGATGGATGA	TTGATGTCTTCGTTAATATAGGT
osteonectin	*on*	ACTAACAACAAGACCTAC	TCCGATGTAATCTATGTG
House keeping gene			
*β-actin*	*actb1*	CCCAAAGCCAACAGAGAGAA	ACCAGAAGCGTACAGAGAGA

## References

[1] Riggs B.L., Khosla S., Melton L.J. (2002). Sex steroids and the construction and conservation of the adult skeleton. Endocr. Rev..

[2] Crockett J.C., Rogers M.J., Coxon F.P., Hocking L.J., Helfrich M.H. (2011). Bone remodelling at a glance. J. Cell Sci..

[3] Feng X., McDonald J.M. (2011). Disorders of bone remodeling. Annu. Rev. Pathol..

[4] Lazner F., Gowen M., Pavasovic D., Kola I. (1999). Osteopetrosis and osteoporosis: Two sides of the same coin. Hum. Mol. Genet..

[5] Benjamin R.M. (2010). Bone health: Preventing osteoporosis. Public Health Rep..

[6] Pisani P., Renna M.D., Conversano F., Casciaro E., Di Paola M., Quarta E., Muratore M., Casciaro S. (2016). Major osteoporotic fragility fractures: Risk factor updates and societal impact. World J. Orthop..

[7] Drake M.T. (2013). Osteoporosis and cancer. Curr. Osteoporos. Rep..

[8] Johnell O., Kanis J.A. (2006). An estimate of the worldwide prevalence and disability associated with osteoporotic fractures. Osteoporos. Int..

[9] Heaney R.P. (2003). Advances in therapy for osteoporosis. Clin. Med. Res..

[10] Bodine P.V., Stauffer B., Ponce-de-Leon H., Bhat R.A., Mangine A., Seestaller-Wehr L.M., Moran R.A., Billiard J., Fukayama S., Komm B.S. (2009). A small molecule inhibitor of the wnt antagonist secreted frizzled-related protein-1 stimulates bone formation. Bone.

[11] Yu P.B., Hong C.C., Sachidanandan C., Babitt J.L., Deng D.Y., Hoyng S.A., Lin H.Y., Bloch K.D., Peterson R.T. (2008). Dorsomorphin inhibits bmp signals required for embryogenesis and iron metabolism. Nat. Chem. Biol..

[12] Brey D.M., Motlekar N.A., Diamond S.L., Mauck R.L., Garino J.P., Burdick J.A. (2011). High-throughput screening of a small molecule library for promoters and inhibitors of mesenchymal stem cell osteogenic differentiation. Biotechnol. Bioeng..

[13] Kim S.N., Bae S.J., Kwak H.B., Min Y.K., Jung S.H., Kim C.H., Kim S.H. (2012). In vitro and in vivo osteogenic activity of licochalcone a. Amino Acids.

[14] Astashkina A., Mann B., Grainger D.W. (2012). A critical evaluation of in vitro cell culture models for high-throughput drug screening and toxicity. Pharmacol. Ther..

[15] Bradaschia-Correa V., Barrence F.A., Ferreira L.B., Massa L.F., Arana-Chavez V.E. (2012). Effect of alendronate on endochondral ossification in mandibular condyles of growing rats. Eur. J. Histochem..

[16] Suster M.L., Kikuta H., Urasaki A., Asakawa K., Kawakami K. (2009). Transgenesis in zebrafish with the tol2 transposon system. Methods Mol. Biol..

[17] Spaink H.P., Cui C., Wiweger M.I., Jansen H.J., Veneman W.J., Marin-Juez R., de Sonneville J., Ordas A., Torraca V., van der Ent W. (2013). Robotic injection of zebrafish embryos for high-throughput screening in disease models. Methods.

[18] Kashii M., Hashimoto J., Nakano T., Umakoshi Y., Yoshikawa H. (2008). Alendronate treatment promotes bone formation with a less anisotropic microstructure during intramembranous ossification in rats. J. Bone Miner. Metab..

[19] Yoshioka T., Okimoto N., Okamoto K., Sakai A. (2013). A comparative study of the effects of daily minodronate and weekly alendronate on upper gastrointestinal symptoms, bone resorption, and back pain in postmenopausal osteoporosis patients. J. Bone Miner. Metab..

[20] Okamoto M., Yamanaka S., Yoshimoto W., Shigematsu T. (2014). Alendronate as an effective treatment for bone loss and vascular calcification in kidney transplant recipients. J. Transplant..

[21] Chen G., Deng C., Li Y.P. (2012). Tgf-beta and bmp signaling in osteoblast differentiation and bone formation. Int. J. Biol. Sci..

[22] Yavropoulou M.P., Yovos J.G. (2014). The role of notch signaling in bone development and disease. Hormones (Athens).

[23] Finkelstein J.S., Hayes A., Hunzelman J.L., Wyland J.J., Lee H., Neer R.M. (2003). The effects of parathyroid hormone, alendronate, or both in men with osteoporosis. N. Engl. J. Med..

[24] Termine J.D., Kleinman H.K., Whitson S.W., Conn K.M., McGarvey M.L., Martin G.R. (1981). Osteonectin, a bone-specific protein linking mineral to collagen. Cell.

[25] Hwang P.-P., Chou M.-Y. (2013). Zebrafish as an animal model to study ion homeostasis. Pflüg. Arch..

[26] Vanoevelen J., Janssens A., Huitema L.F., Hammond C.L., Metz J.R., Flik G., Voets T., Schulte-Merker S. (2011). Trpv5/6 is vital for epithelial calcium uptake and bone formation. FASEB J..

[27] Pan T.-C., Liao B.-K., Huang C.-J., Lin L.-Y., Hwang P.-P. (2005). Epithelial Ca^2+^ channel expression and Ca^2+^ uptake in developing zebrafish. Am. J. Physiol.-Regul. Integr. Comp. Physiol..

[28] Olsen B.R., Reginato A.M., Wang W. (2000). Bone development. Annu. Rev. Cell Dev. Biol..

[29] Akiyama H., Kim J.E., Nakashima K., Balmes G., Iwai N., Deng J.M., Zhang Z., Martin J.F., Behringer R.R., Nakamura T. (2005). Osteo-chondroprogenitor cells are derived from sox9 expressing precursors. Proc. Natl. Acad. Sci. USA.

[30] Fleming A., Keynes R., Tannahill D. (2004). A central role for the notochord in vertebral patterning. Development.

[31] Mabee P.M., Noordsy M. (2004). Development of the paired fins in the paddlefish, polyodon spathula. J. Morphol..

[32] Flores M.V., Tsang V.W., Hu W., Kalev-Zylinska M., Postlethwait J., Crosier P., Crosier K., Fisher S. (2004). Duplicate zebrafish runx2 orthologues are expressed in developing skeletal elements. Gene Expr. Patterns.

[33] Fisher S., Halpern M.E. (1999). Patterning the zebrafish axial skeleton requires early chordin function. Nat. Genet..

[34] Melton L.J., Johnell O., Lau E., Mautalen C.A., Seeman E. (2004). Osteoporosis and the global competition for health care resources. J. Bone Miner. Res..

[35] Fleming A., Sato M., Goldsmith P. (2005). High-throughput in vivo screening for bone anabolic compounds with zebrafish. J. Biomol. Screen..

[36] Tarasco M., Laizé V., Cardeira J., Cancela M.L., Gavaia P.J. (2017). The zebrafish operculum: A powerful system to assess osteogenic bioactivities of molecules with pharmacological and toxicological relevance. Comp. Biochem. Physiol. Part C Toxicol Pharmacol..

[37] Westerfield M. (1995). The Zebrafish Book: A Guide for the Laboratory Use of Zebrafish (Brachydanio Rerio).

[38] Du S.J., Frenkel V., Kindschi G., Zohar Y. (2001). Visualizing normal and defective bone development in zebrafish embryos using the fluorescent chromophore calcein. Dev. Biol..

[39] Schmittgen T.D., Livak K.J. (2008). Analyzing real-time pcr data by the comparative ct method. Nat. Protoc..

